# The Communication Factor EDF and the Toxin–Antitoxin Module *mazEF* Determine the Mode of Action of Antibiotics

**DOI:** 10.1371/journal.pbio.0060319

**Published:** 2008-12-16

**Authors:** Ilana Kolodkin-Gal, Boaz Sat, Alex Keshet, Hanna Engelberg- Kulka

**Affiliations:** Department of Molecular Biology, The Hebrew University-Hadassah Medical School, Jerusalem, Israel; Harvard University, United States of America

## Abstract

It was recently reported that the production of Reactive Oxygen Species (ROS) is a common mechanism of cell death induced by bactericidal antibiotics. Here we show that triggering the Escherichia coli chromosomal toxin–antitoxin system *mazEF* is an additional determinant in the mode of action of some antibiotics. We treated E. coli cultures by antibiotics belonging to one of two groups: (i) Inhibitors of transcription and/or translation, and (ii) DNA damaging. We found that antibiotics of both groups caused: (i) *mazEF*-mediated cell death, and (ii) the production of ROS through MazF action. However, only antibiotics of the first group caused *mazEF*-mediated cell death that is ROS-dependent, whereas those of the second group caused *mazEF*-mediated cell death by an ROS-independent pathway. Furthermore, our results showed that the mode of action of antibiotics was determined by the ability of E. coli cells to communicate through the signaling molecule Extracellular Death Factor (EDF) participating in *mazEF* induction.

## Author Summary

The modes of action of antibiotics are mainly characterized by the effect they have on their targets. Recently, it was reported that the formation of Reactive Oxygen Species (ROS) is a common downstream mechanism of antibiotics that leads to cell death—called bactericidal—while the bacteriostatic antibiotics—causing growth arrest—do not cause ROS formation. We uncovered complexity in how an antibiotic kills by linking antibiotic action and ROS formation with the bacterial toxin–antitoxin system called *mazEF*, a system by which bacteria are known to commit suicide. We show that *mazEF* affects antibiotics cell death through ROS-dependent and ROS-independent mechanisms. Following antibiotic treatment, the communication signaling peptide, called Extracellular Death Factor (EDF), mediates cell death through the activation of the *mazEF* system. Our study challenges the classical division between bacteriostatic and bactericidal antibiotics and provides evidence that antibiotics' mode of action is determined by the ability of the bacteria to communicate through the signaling peptide EDF.

## Abstract 3

The mode of action of certain antibiotics is revealed in a study using different strains of*E. coliM*, which implicates*mazEF*-mediated cell death and the formation of reactive oxygen species.

## Introduction

Traditionally, antibiotics are classed as either “bactericidal,” meaning that they can kill bacteria, or “bacteriostatic,” meaning that they can only inhibit bacterial growth. Bacteriostatic drugs may be effective, because inhibiting bacterial growth allows the body's defence mechanisms to eliminate the pathogenic bacteria [1]. The mechanisms of antibiotics actions were well studied, particularly in relation to their targets interactions. Accordingly, they fall into three main groups: DNA damage-causing agents, inhibitors of protein synthesis, and inhibitors of cell wall turnover [2]. Recently, a downstream common mechanism of antibiotics leading to cell death has been reported. It was shown that the three major groups of bactericidal antibiotics, regardless of their targets interactions, stimulate the production of hydroxyl radicals in Gram-negative and Gram-positive bacteria, which ultimately causes cell death. In contrast, the bacteriostatic antibiotics do not produce hydroxyl radicals [3].

We have previously shown that some antibiotics trigger cell death by the activation of the built-in death system *mazEF* of Escherichia coli [4–6]. *mazEF* is a toxin–antitoxin (TA) module found on the chromosomes of many bacteria, including pathogens [7–10]. *E. coli mazF* specifies for the stable toxin MazF, and *mazE* specifies for the labile antitoxin, MazE. In vivo, MazE is degraded by the ATP-dependent ClpAP serine protease [11]. MazF is a sequence-specific endoribonuclease that preferentially cleaves single-stranded mRNAs at ACA sequences [12,13], and thereby inhibits translation [12,14]. MazE counteracts the action of MazF. Because MazE is a labile protein, preventing MazF-mediated action requires the continuous production of MazE. Thus, any stressful condition that prevents the expression of the chromosomally borne *mazEF* module will lead to the reduction of MazE in the cell, permitting the toxin MazF to act freely. Such stressful conditions can be caused by antibiotics, including (i) those that inhibit transcription and/or translation like rifampicin, chloramphenicol, and spectinomycin [4]; and (ii) those that cause DNA damage like mitomycin C, nalidixic acid, and trimethoprim [6,15–17]. Each of these antibiotics is well known to cause bacterial cell death [18,19]. It is obvious that antibiotics belonging to the first group prevent *mazEF* expression. As for antibiotics belonging to the second group, we have shown that thymine starvation by tirmethoprim inactivates the major promoter P_2_ of *mazEF* [5], and we have speculated that this inactivation may be caused indirectly by the induction of ppGpp synthesis, known to inhibit the *mazEF* P_2_ promoter [11], and/or by some specific protein(s) that could sense the damage to the DNA. The nature of the mechanism that is involved in *mazEF* P_2_ promoter inactivation by trimethoprim, and whether it is involved by other DNA-damaging antibiotics, is still unknown. In addition, we recently reported that *E. coli mazEF*-mediated cell death is a population phenomenon requiring a communication signaling molecule that we call the Extracellular Death Factor (EDF) [20]. EDF is the linear penta-peptide NNWNN. Each of the five EDF amino acids is important for its *mazEF*-mediated killing activity, and the terminal asparagines are the most crucial. EDF production involves the Glucose-6-phosphate dehydrogenase, Zwf, and the protease ClpXP [20,21].

Here we asked: Does the action of *mazEF-*mediated cell death in E. coli involve the production of Reactive Oxygen Species (ROS)? We treated E. coli cultures with one of two groups of antibiotics: (i) inhibitors of transcription and/or translation, traditionally considered as being bacteriostatic; and (ii) DNA-damaging agents, traditionally considered as being bactericidal. We found that antibiotics belonging to both groups caused: (i) *mazEF*-mediated cell death; and (ii) the production of ROS, which is generated through the action of MazF. However, although both groups of antibiotics caused ROS production, only antibiotics belonging to the first group caused *mazEF*-mediated cell death that is ROS-dependent. In contrast, antibiotics belonging to the second group caused a *mazEF*-mediated cell death pathway that is ROS-independent. Thus, our results suggest that there are at least two *mazEF*-mediated cell death pathways: (i) ROS-dependent; and (ii) ROS-independent. Furthermore, our results indicate that the mode of action of antibiotics was a function of the ability of E. coli cells to communicate with each other through the signaling molecule EDF.

## Results

### ROS-Detoxifying Enzymes Prevented *mazEF*-Mediated Cell Death Induced by the Inhibition of Transcription and/or Translation

In every aerobic organism, respiration results in the formation of ROS, including hydrogen peroxide, superoxide anion, and hydroxyl radicals. Theses ROS have been implicated in programmed cell death in eukaryotes [22,23], including yeast [24,25], and in the action of some antibiotics [3]. Cells have developed mechanisms for detoxifying ROS and for repairing oxidative damage [26–30].

**Figure 1:**
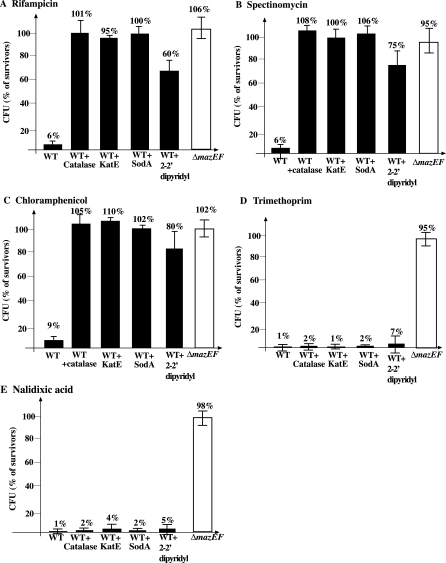
Catalase or Superoxide Dismutase Prevented *E. coli mazEF-*Mediated Cell Death Induced by Inhibitors of Transcription and/or Translation E. coli MC4100*relA* ^+^ (WT), MC4100*relA* ^+^/pQE*katE* (WT+KatE), MC4100*relA* ^+^/pQE*sodA* (WT+SodA) or MC4100*relA* ^+^Δ*mazEF* (Δ*mazEF*) were grown aerobically to mid-log phase. Cells were incubated without shaking at 37 °C for 10 min, followed by the addition of: (A) rifampicin (20 μg/ml) for 10 min; (B) spectinomycin (1 mg/ml) for 1 h; (C) chloramphenicol (50 μg/ml) for 20 min; (D) trimethoprim (2 μg/ml) for 1h; and (E) nalidixic acid (1 mg/ml) for 10 min. The wild type strain (WT) is represented by black bars and the derivative Δ*mazEF* strain is represented by white bars. Error bars indicate standard deviation.

Rifampicin, through its action on the β subunit of E. coli RNA polymerase [19], prevents the transcription of *mazEF*, thereby leading to *mazEF-*mediated cell death [4]. In these previous experiments, we have shown that when *mazEF* is transiently induced, wild-type cells die whereas the Δ*mazEF* derivative does not die. Here, using similar inducing agents and experimental conditions, we found that *mazEF*-mediated cell death is prevented by adding catalase from outside the cells or by over-expression of catalase or superoxide dismutase (Figure 1). These enzymes are well known to detoxify ROS or to inhibit the formation of ROS [26–30]. Previously, we showed that *mazEF*–mediated cell death is triggered by DNA-damaging agents [5,6] including (i) nalidixic acid, an inhibitor of the topoisomerase gyrase [31], and (ii) trimethoprim, an inducer of thymine starvation [18]. The latter was shown to inhibit transcription from the promoter P_2_ of *mazEF* [5]. Here we found that, in contrast to *mazEF*-mediated cell death induced by inhibitors of transcription (Figure 1A) or translation (Figure 1B and 1C), when *mazEF* was induced by the DNA-damaging agents trimethoprim (Figure 1D) or nalidixic acid (Figure 1E), *mazEF*-mediated cell death was not prevented by the presence of catalase or superoxide dismutase. In these cases, even increasing the amounts of the ROS-detoxifying enzymes by about five times did not prevent *mazEF*-mediated cell death (unpublished data).

2–2′-dipyridyl is an iron chelator that prevents the Fenton-mediated hydroxyl radical formation [32]. A knockout of *iscS* substantially impairs iron sulfur cluster synthesis capability and therefore it prevents the Fenton reaction [33,34]. Here we show that similarly to catalase and superoxide dismutase, 2–2′-dipyridyl (Figure 1) and a knockout of *iscS* (Figure S1) prevented *mazEF*-mediated cell death triggered by inhibitors of transcription and/or translation, but not by antibiotics causing DNA damage.

We further confirmed that rifampicin is bactericidal due to its action through the *mazEF*-mediated cell death network, which takes place via oxidative pathways. We show that in E. coli strain MC4100, deleted for the important TCA-cycle component genes—either *icdA* (coding for isocitrate dehydrogenase A) or *acnB* (coding for aconitase B) cell death induced by rifampicin was prevented (Figure S2).

Thus, our results show that ROS-detoxifying enzymes prevent *mazEF*-mediated cell death induced by the inhibition of transcription and/or translation, but not by DNA damage.

### Activation of *E. coli mazEF* by Some Antibiotics Increases the Level of Protein Oxidation (Carbonylation)

**Figure 2:**
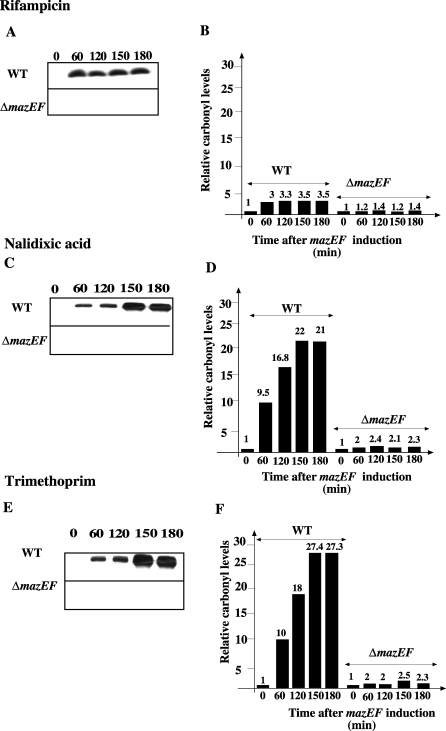
*mazEF*-Mediated Carbonylation of Cellular Protein Following Treatment by Various Antibiotics (A, C, and E) Protein carbonylation. E. coli strains MC4100*relA* ^+^ (WT) and its derivative MC4100*rel A* ^+^Δ*mazEF* (Δ*mazEF*) were grown in aerobic conditions. Logarithmic cells were pre-incubated as in Figure 1, and treated with antibiotics as follows: (A) rifampicin (20 μg/ml) for 10 min; (C) nalidixic acid (1 mg/ml) for 10 min; (E) trimethoprim (2 μg/ml) for 1h. Protein carbonylation was determined. (B, D, and F) Relative carbonyl levels of a representative experiment is described in (A, C, and E). The intensity of bands obtained in same time points as in panels (A, C, and E) from three independent experiments was quantified. The numbers express the relative carbonyl levels of each treated strain compared to untreated WT strain.

Having found that ROS-detoxifying enzymes prevented *mazEF*-mediated cell death (Figures 1A–1C), we expected that triggering the *mazEF* module would induce ROS formation that would be reflected in the cellular level of protein carbonylation. To detect oxidized proteins carrying carbonyl groups, we used the immunochemical assay from the Chemicon Oxyblot Kit. The triggering of *mazEF* was carried out in E. coli MC4100*relA* ^+^ (wild type (WT)) with rifampicin (Figure 2A and 2B), or nalidixic acid (Figure 2C and 2D) for 10 min, or trimethoprim (Figure 2E and 2F) for 1 h. After removing the antibiotics, we prepared crude protein extracts from the treated cultures. Activating *mazEF* in WT cells by each of these three antibiotics led to an increase in the level of protein carbonylation within 60 min (Figure 2). Under the same conditions, we observed no such change in the very low basal level of carbonylation in the Δ*mazEF* derivative strain (Figure 2 and Figure S3). Furthermore, when we quantified the relative levels of protein carbonylation, we found that the DNA-damaging agents nalidixic acid and trimethoprim each induced significantly higher levels of *mazE*F-dependent carbonylation than did the transcription inhibitor rifampicin. For example, after the addition of trimethoprim, the relative carbonyl level increased by 27 times (Figure 2F), but after the addition rifampicin, it increased only by about 3.5 times (Figure 2B). Note also that, while in the case of rifampicin, *mazEF*-dependent carbonylation remained constant after 1 h (Figure 2A and 2B), in the case of DNA damage, a gradual increase in the level of carbonylation from 1 to 3 h was observed (Figure 2C–2F).

### Catalase Prevented *mazEF*-Dependent Protein Carbonylation

We found that adding or causing the overproduction of catalase completely prevented *mazEF*-dependent protein carbonylation (Figure S4A–S4E). When we induced *mazEF* with rifampicin, adding as little as 20 μg/ml of catalase was sufficient to prevent protein carbonylation (Figure S4A and S4B). However, when we induced *mazEF* with trimethoprim, five times more catalase (100 μg/ml) was required to completely prevent protein carbonylation (Figure S4D and S4E). This increased requirement seemed reasonable, since much more carbonylation resulted from activating *mazEF* by trimethoprim (Figure S4E) or nalidixic acid (Figure 2C and 2D) than by rifampicin (Figure 2A and 2B). Based on these results, we asked: would the overproduction or the addition of catalase at concentrations that prevented *mazEF*-dependent carbonylation also prevent *mazEF*-mediated cell death? This clearly occurs when *mazEF* is induced by rifampicin (Figure S4C), however, not in the case of the DNA-damaging agent trimethoprim (Figure S4F). Here, although high concentrations of catalase (100 μg/ml) were sufficient to prevent *mazEF*-dependent carbonylation (Figure S4D and S4E), they could not prevent cell death (Figure S4F).

**Figure 3:**
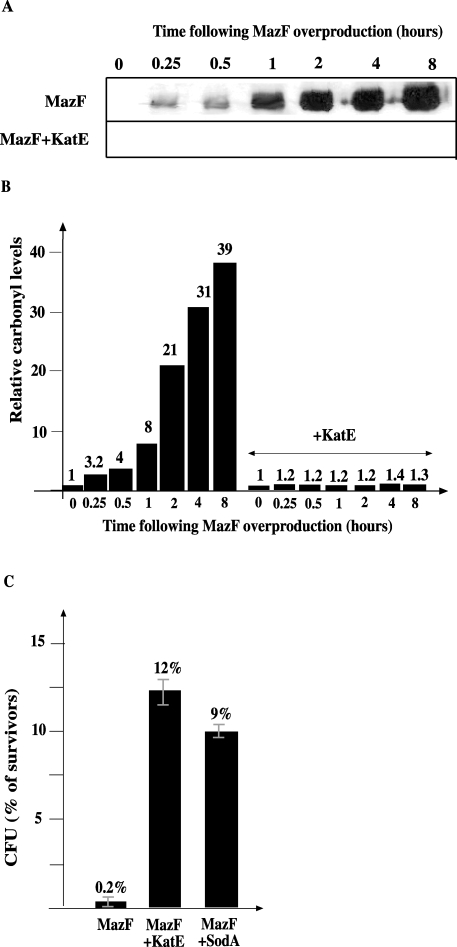
MazF Overproduction Induces Cellular Protein Carbonylation That is Prevented by Catalase E. coli strains MC4100*relA* ^+^/pBAD-*mazF* (MazF), MC4100*relA* ^+^/pQE*katE*/pBAD-*mazF* (MazF+KatE), and MC4100*relA* ^+^/pQE*sodA/* pBAD-*mazF* (MazF+SodA) were grown in M9 minimal medium and MazF was induced. (A) Protein carbonylation; (B) relative carbonyl levels described in (A). The intensity of each band presented in (A) quantified as described in the legend for Figure 2. (C) Percent (%) of survivors was determined 8 h following the induction of *mazF* expression.

Then we asked: would artificially overproducing MazF induce carbonylation? If so, in cultures in which MazF were artificially overproduced, would preventing carbonylation by increasing the concentration of catalase prevent cell death by MazF? We used a plasmid-borne *mazF* gene under the regulation of the *E. coli araBAD* promoter, permitting the expression of *mazF* by the addition of arabinose to the cells and its repression by the addition of glucose [35]. We found that, over a period of 15–120 min, overproducing MazF led to a gradual increase in the level of carbonylated proteins (Figure 3A and 3B). Causing the overexpresseion of *katE*, the gene for catalase, or *sodA*, the gene for superoxide dismutase (unpublished data), before MazF was overproduced completely prevented protein carbonylation (Figure 3B). However, although overproducing of either KatE or SodA led to significant increases in cell survival (about 60 times), MazF-mediated cell death was not completely prevented (Figure 3C).

Thus, elevated levels of ROS are produced by either inducing *mazEF* by stressful conditions, or by the overproduction of MazF. However, cell death is only completely prevented by catalase when *mazEF* is induced by stressful conditions that inhibit transcription and /or translation.

### Completely Anaerobic Growth Conditions Prevented *mazEF*-Mediated Cell Death When *mazEF* Was Activated by Inhibiting Transcription and/or Translation but Not When Activated by DNA Damage

Mistranslated proteins are substrates for carbonylation [36]. Because MazF is an endoribonuclease that cleaves mRNAs containing ACA sequences [12,13], we hypothesized that the action of MazF could produce mistranslated proteins that could then be substrates for carbonylation. We wished to distinguish if the effect of MazF on protein carbonylation (Figure 3) that we have reported here could be attributed to the formation of ROS or to the mis-translation of proteins. Under conditions of completely anaerobic growth, we expected that mistranslation would still occur but ROS would not be formed.

**Figure 4:**
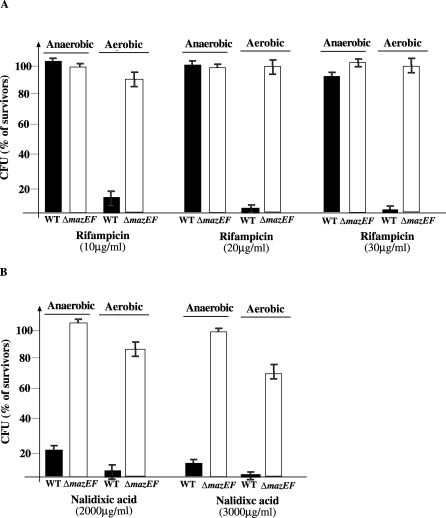
Completely Anaerobic Growth Conditions Prevented *mazEF*-Mediated Cell Death When *mazEF* Was Activated by Inhibiting Transcription and/or Translation but Not When Activated by DNA Damage E. coli MC4100*relA* ^+^ (WT) and MC4100*relA* ^+^Δ*mazEF* (Δ*mazEF*) were grown under either aerobic or anaerobic conditions. Cells were incubated either aerobically, without shaking, or in an anaerobic jar with: (A) rifampicin (10, 20, or 30 μg/ml) for 10 min or (B) nalidixic acid (2–3 mg/ml) for 10 min.

To avoid the production of ROS, we studied *mazEF*-mediated cell death triggered by various antibiotics under completely anaerobic conditions, comparing those results to those of cultures grown aerobically. We activated *mazEF* by adding rifampicin to inhibit transcription (Figure 4A), by adding spectinomycin to inhibit translation (unpublished data), or by adding nalidixic acid to cause DNA damage (Figure 4B). Under conditions of aerobic growth, we observed *mazEF*-mediated cell death when the *mazEF* module was activated by rifampicin at concentrations between 10 and 30 μg/ml (Figure 4A). When we added the same (10–30 μg/ml, Figure 4A) or even higher concentrations (up to 60 μg/ml, unpublished data) of rifampicin to WT cells grown anaerobically, *mazEF*-mediated cell death was completely prevented. We observed similar results under anaerobic conditions when *mazEF* was activated by spectinomycin (unpublished data). However, when the *mazEF* module was activated by nalidicxic acid (2–3 mg/ml), we observed *mazEF-*mediated cell death even under anaerobic growth conditions (Figure 4B). Thus, in respect to cell death, inhibitors of transcription and/or translation behave differently than DNA damaging agents.

When MazF is overproduced under anaerobic conditions (Figure S5): (i) ROS is not produced, further indicating that ROS is not generated due to mistranslation; and (ii) MazF still mediates cell death, although in a much less extend than under aerobic conditions. This result further support our model on the existence of a MazF/ ROS-independent form of cell death (see Discussion).

### Communication Factor EDF and *mazEF* Determine the Mode of Action of Some Antibiotics

#### EDF and *mazEF* cause the inhibitor of transcription antibiotic, rifampicin, to be bacteriocidal through the formation of ROS.

**Figure 5:**
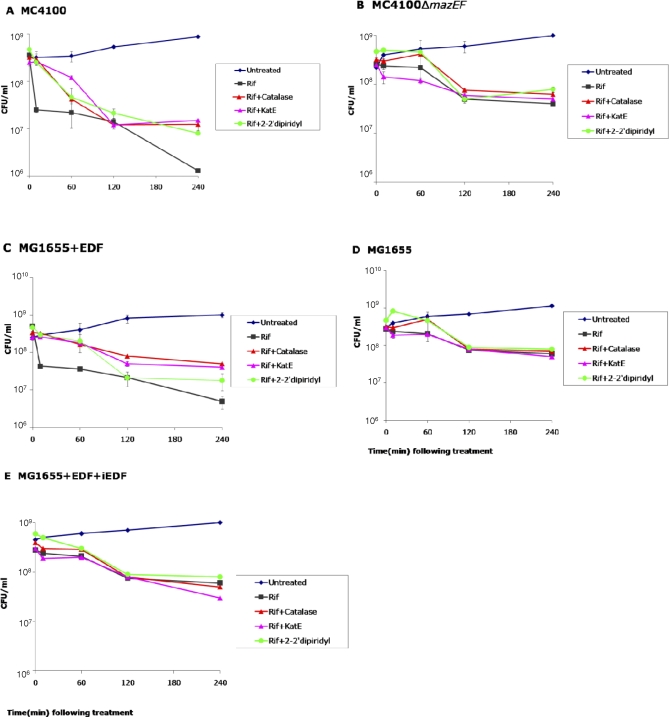
EDF and *mazEF* Are Important Determinants in the Mode of Action of Rifampicin in E. coli Logarithmic changes in the number of colony forming units per milliliter (CFU/ml) following exposure to rifampicin (20 μg/ml) in E. coli strains: (A) MC4100*relA* ^+^; (B) MC4100*relA* ^+^Δ*mazEF*; (C) MG1655 + EDF (0.05 μg/ml); (D) MG1655; and (E) MG1655 + EDF (0.05 μg/ml) + iEDF (0.05 μg/ml).

We previously reported that the signal molecule EDF participates in *mazEF* induction [20]. Here we asked: does the ability of E. coli cells to communicate through the participation of EDF in *mazEF* induction determine whether an antibiotic will be bacteriostatic or bacteriocidal? For this purpose, we grew E. coli cells in M9 medium until mid-log (growth conditions which we found as being optimal for the induction of the EDF-*mazEF* system). Subsequently, the cells were treated with rifampicin (Figure 5) and cell viability was determined during 4 hours. We used three E. coli strains: (i) E. coli MC4100*relA^+^*; (ii) E. coli MC4100*relA^+^*Δ*mazEF*; and (iii) E. coli MG1655. Comparing the effects of rifampicin on the viability of E. coli MC4100*relA* ^+^ and its Δ*mazEF* derivative revealed a short-term effect and a long-term *mazEF-*mediated effect (Figure 5A and 5B). Treating with low concentrations (20μ g/ml) of rifampicin for as little as 10 min resulted in *mazEF*-mediated cell death (90% decrease in viability), which was prevented by the presence of the ROS detoxifying enzyme catalase or by the iron chelator 2–2′-dipyridyl (Figure 5A). Prolonging the time of exposure to rifampicin up to 4 h led to an increase of an additional order of magnitude in *mazEF*-mediated cell death. The presence of catalase or 2–2′-dipyridyl only partially prevented this long-term effect (Figure 5A). Thus, rifampicin was bactericidal for strain MC4100*relA* ^+^ (Figure 5A), but bacteriostatic for strain MC4100*relA* ^+^Δ*mazEF*, for which we observed no effect upon the addition of catalase or 2–2′-dipyridyl (Figure 5B).

In contrast, our results revealed that rifampicin was bacteriostatic for strain MG1655 (Figure 5D), even though MG1655 bears *mazEF* on its chromosome [37]. These results confirm those of Kohanski and colleagues [3], who reported that rifampicin was bacteriostatic for strain E. coli MG1655. We have recently reported that, unlike MC4100*relA^+^* and several other well studied E. coli strains, MG1655 is partially defective both in the production of EDF and in the response to it. Thus, in strain MG1655, *mazEF* is activated (and thereby cell death occurs) only by the addition of high concentrations of EDF [21]. Therefore, we here added the appropriate concentrations of synthetic EDF to the culture of MG1655 and studied the mode of action of rifampicin. We found that rifampicin was bacteriostatic for untreated MG1655 cultures (Figure 5D) and bactericidal in the presence of added synthetic EDF (Figure 5C). In particular, for strain MC4100*relA* ^+^ (Figure 5A) and strain MG16565 with added synthetic EDF (Figure 5C), rifampicin was bactericidal: within 10 min after the addition of rifampicin, we observed *mazEF*-mediated cell death (90% decrease in viability). This level of cell death was prevented by the addition of the ROS detoxifying enzyme catalase or its internal over-expression or the addition of iron chelator 2–2′-dipyridyl (Figure 5A and 5C). Prolonging the time of exposure to rifampicin up to 4 h led to an increase of at least another order of magnitude in *mazEF*-mediated cell death, which was only partially prevented by these ROS detoxifying elements (Figure 5A and 5C). For both strains MC4100*relA^+^*Δ*mazEF* (Figure 5B) and MG1655 without added EDF (Figure 5D), the effect of the addition of rifampicin was bacteriostatic and was not affected by the addition of catalase or its over-expression or 2–2′-dipyridyl. Thus, the bactericidal effect of rifampicin, which acted through the formation of ROS, was dependent on both *mazEF* and EDF.

We confirmed the role of EDF in the bactericidal action of rifampicin by using iEDF, an inhibitor of EDF in which the first amino acid aspargine of EDF has been changed to glycine [20]. In these experiments, we added EDF and iEDF to a culture of MG1655. As shown, the addition of iEDF caused a bacteriostatic action of rifampicin (Figure 5E). Note that the production and activity of EDF were apparent only when the cell cultures were grown in minimal medium M9, and not in rich LB medium (unpublished data).

#### EDF and *mazEF* cause the DNA-damaging antibiotic, nalidixic acid, to be bactericidal through ROS-independent pathway(s).

**Figure 6:**
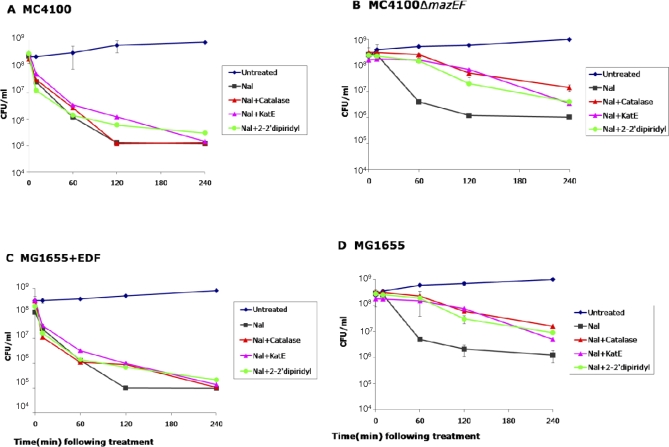
EDF and *mazEF* Are Important Determinants in the Mode of Action of Nalidixic Acid in E. coli Logarithmic changes in the number of colony forming units per milliliter (CFU/ml) following exposure to nalidixic acid (1 mg/ml) in E. coli strains: (A) MC4100r*elA* ^+^; (B) MC4100*relA* ^+^Δ*mazEF*; (C) MG1655 + EDF (0.05 μg/ml); and (D) MG1655 .

We also studied whether EDF and *mazEF* are determinants in the mode of action of the DNA-damaging antibiotic nalidixic acid (Figure 6). Here, the same experimental conditions and strains were applied as in our experiments with rifampicin (Figure 5). As in the case of rifampicin, we found that nalidixic acid (1 mg/ml) had both short- and long-term effects on the viability of E. coli MC4100*relA^+^* and its Δ*mazEF* derivative (Figure 6A and 6B). In contrast to rifampicin, exposure to nalidixic acid for 10 min resulted in *mazEF*-mediated cell death, which was not neutralized by the addition of catalase or 2–2′-dipyridyl (Figure 6A and 6B). However, in contrast to the short exposure effect, upon exposure to nalidixic acid for a long period of time (4 h), a *mazEF*-independent death was observed; by 60 min of exposure, the level of cell death had increased by another order of magnitude in MC4100*relA* ^+^ as well as in its Δ*mazEF* derivative (compare Figure 6A and 6B). This long- term effect was partially prevented by the addition of catalase or 2–2′-dipyridyl in Δ*mazEF* derivative of MC4100*relA^+^* (Figure 6B), and in strain MG1655 that is defective in EDF production and response [37] (Figure 6D). Such a cell death phenomenon is *mazEF-*independent and ROS-dependent.

Our results in MG1655 confirmed these of Kohanski et al. [3] in the same strain. However, we also show that the addition of synthetic EDF to the culture of E. coli strain MG1655 induces the *mazEF*-dependent short- and long-term effect of nalidixic acid (Figure 6C). This death pathway is not prevented by the ROS detoxifying enzyme catalase or 2–2′-dipyridyl (Figure 6C), indicating that EDF enables the *mazEF*-mediated death pathway that is activated by DNA damage to take over (see Discussion).

In summary, our results revealed that the mode of action of some antibiotics is determined by the ability of E. coli cells to communicate through the signaling molecule EDF and the presence of the chromosomal *mazEF* module.

## Discussion

Here we report on two important findings related to antibiotics and the built-in death system of E. coli, EDF-*mazEF*. In the first, we show that *mazEF* mediates at least two cell death pathways: (i) ROS-dependent; and (ii) ROS-independent. In the second we show that EDF-*mazEF* determines the mode of action of some antibiotics in E. coli. We will discuss each of these topics separately.

### *E. coli mazEF* Mediates at Least Two Cell Death Pathways: (i) ROS-Dependent and (ii) ROS-Independent

No direct method is available to quantify intracellular levels of ROS. A method recently used by Collins and colleagues [3,38] is based on derivatized fluoresceins, and was recently criticized [39]. Here, we used a different method in which we detected oxidized proteins carrying carbonyl groups. Protein carbonylation is caused by ROS [40–42]. In E. coli, we found that overproducing MazF (Figure 3), or transiently activating chromosomal *mazEF* by various antibiotics (Figure 2), led to significant increases in the levels of oxidized (carbonylated) proteins. Therefore, the endoribonuclease MazF that produces truncated proteins, including ROS-detoxifying enzymes, can lead to an increase in the level of ROS and thereby to an increase in the level of protein carbonylation. Here we have offered two lines of evidence supporting that ROS formation is involved in *mazEF*-mediated cell death, at least under conditions of aerobic growth and when activated by the inhibition of transcription or translation. (i) The ROS detoxifying enzymes catalase or superoxide dismutase, and the iron chelator 2–2′-dipyridyl completely prevented death when they were induced before the activation of *mazEF* by antibiotics that inhibit transcription and/or translation (Figure 1). (ii) We observed similar results when *mazEF* was activated under completely anaerobic growth conditions (Figure 4). Thus, we found that when cell death was activated by the inhibition of transcription and/or translation, *mazEF-*mediated cell death was ROS-dependent. In contrast, it was clear that, when induced by DNA damage, *mazEF*-mediated cell death was independent of ROS since cell death was not prevented by the presence of ROS detoxifying enzymes or the iron chelator 2–2′-dipyridyl (Figure 1C and 1D) or by anaerobic conditions (Figure 4).

**Figure 7:**
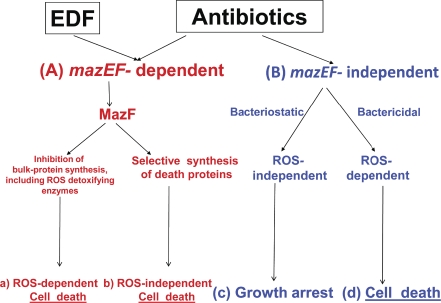
Alternative Death Pathways Induced by Some Antibiotics in E. coli (A) Antibiotic induction of EDF-*mazEF* activates MazF (in red). (a) Antibiotics that inhibit transcription and/or translation cause cell death that is ROS-dependent, probably by inhibition of bulk-protein synthesis, including that of ROS detoxifying enzymes. (b) In contrast, antibiotics causing DNA damage trigger an ROS-independent death pathway(s), probably by the selective synthesis of death proteins. (B) Antibiotic induction of EDF-*mazEF*-independent death pathways (in blue). (c) Antibiotics that inhibit transcription and/or translation do not cause ROS production. Therefore, they do not kill the cells but cause growth arrest, and thus are bacteriostatic. (d) In contrast, antibiotics that cause DNA damage do induce ROS production, and thereby lead to cell death.

Based on our results, we suggest that antibiotic-induced *mazEF*-mediated cell death is a developmental process for which there are at least two pathways: ROS-dependent (Figure 7A pathway (a)) and ROS-independent (Figure 7A pathway (b)). The ROS-dependent *mazEF*-mediated cell death takes place when *mazEF* is induced by inhibitors of transcription or translation. Under those conditions, cell death is prevented either by anaerobic conditions (Figure 4A) or by ROS detoxifying enzymes or the iron chelator (Figure 1A–1C). MazF due to its endoribonucleolytic effect inhibits bulk protein synthesis, including ROS detoxifying enzymes, thereby elevated levels of ROS are produced leading to cell death.Therefore, when *mazEF* is activated by the inhibition of transcription or translation, ROS, and not MazF, is the mediator of cell death (Figure 7A pathway (a)). The ROS-independent pathway takes place when *mazEF* is activated by agents causing DNA damage. Under those conditions, we found that although high levels of ROS were formed (Figure 2D and 2E), neither catalase nor superoxide dismutase, nor anaerobic conditions prevented cell death (Figure 4). We suggest that when certain antibiotics cause damage to the DNA, the endoribonucleolytic action of MazF triggers a downstream cascade leading to cell death. That such a downstream cascade exists is supported by our results showing that MazE can reverse MazF toxicity only during a short window of time [17,35]. Moreover, our recent experiments have shown that although MazF leads to the inhibition of synthesis of most E. coli proteins, it still permits the synthesis of a small group of proteins that participate in cell death (Amitai S, IK-G, Hananya-Meltabashi M, Sacher A, HE-K, unpublished data). This downstream cascade might be initiated by a special mechanism through which MazF would cleave mRNAs at specific sites [12,13] leading to the selective synthesis of proteins encoded by mRNAs that are resistant to the cleavage of MazF. Our results suggest that such proteins may function as executioners of cell death independent of ROS, possibly by acting more rapidly or efficiently than does ROS (Amitai S, IK-G, Hananya-Meltabashi M, Sacher A, HE-K, unpublished data) (Figure 7A pathway (b)).

In addition, our recent experiments also show that inducing *mazEF* by inhibitors of transcription and/or translation also leads to a selective synthesis of “death” proteins acting downstream to MazF, which are different from these induced by DNA damage (Amitai S, IK-G, Hananya-Meltabashi M, Sacher A, HE-K, unpublished data). The “death” proteins induced by inhibitors of transcription and/or translation are probably less potent in the death process than these induced by DNA damage, and therefore in order to induce cell death, they may have to act in combination with ROS.

The level of ROS produced by inhibitors of transcription and or translation is about 10 times lower than that obtained by DNA damaging agents (Figure 2). Therefore, though blocking translation by antibiotics would increase the level of ROS, the threshold of ROS obtained may be too low to enable cell death (by itself) and may still require the assistance of the death proteins that are selectively synthesized by MazF.

### Communication Factor EDF and *mazEF* as Determinants in the Mode of Action of Some Antibiotics in E. coli

It is well known that there are two classes of antibiotics: (i) bacteriostatic, including inhibitors of transcription and or translation, and (ii) bactericidal, including DNA damage agents [1]. Recently, Kohanski and colleagues have reported that bactericidal antibiotics lead to the production of ROS, and thereby cell death, but that bacteriostatic antibiotics do not [3]. However, the experiments on which they based their report were carried out in E. coli strain MG1655, which is defective in the production of the communication factor EDF [21] that is required for the activation of *E. coli mazEF* [20]. Here we show that antibiotics can induce several alternative pathways leading to cell death (Figure 7), and the communication factor EDF determines if the *mazEF*-mediated pathways take over (Figure 7A pathways (a) and (b)). We found that the addition of synthetic EDF to strain MG1655 switches the culture from a *mazEF*-independent growth arrest (Figure 5B and 5D), or *mazEF*-independent cell death (Figure 6B and 6D) into a *mazEF*- dependent cell death (Figures 5A, 5C, 6A, and 6C). Under the experimental conditions in which the EDF-*mazEF*–mediating pathways operated, the transcriptional and translational inhibitor rifampicin, traditionally known as being bacteriostatic, became bactericidal when it activated the EDF-*mazEF* system (Figure 5). The free MazF led to the production of ROS, which led to ROS-dependent death. Antibiotics causing DNA damage, like nalidixic acid, also activated the EDF-*mazEF* system so that the free MazF caused ROS production (Figure 2). But in this case, cell death was ROS-independent (Figure 6). We suggest that the ROS-independent pathway requires a selective production of proteins whose mRNA are resistant to the action of MazF [15]. We have also shown that during a long period of treatment with antibiotics causing DNA damage, an EDF-*mazEF*–independent cell death takes place which is ROS-dependent (Figures 6 and 7B pathway (d)). We suggest that this was actually the mode of action of DNA-damaging antibiotics reported by Collins and colleagues [3,38]. Until recently, the modes of action of antibiotics were studied mainly as a function of their target of interactions [2]. Kohanski and colleagues [3] were the first to show that ROS formation is a common mechanism downstream of the action antibiotics, and that it is the ROS that lead to cell death. Our work has revealed yet another dimension to the mode of action of some antibiotics. We found that it is determined by the ability of E. coli cells to communicate through the signaling molecule EDF as it participates in *mazEF* induction. Thus, using synthetic EDF together with an antibiotic that is an inhibitor of transcription or translation could lead to an increase in the efficiency of killing the bacterial cells, even in the initial stage of infection when the density of the bacteria is low. This implies that EDF (or its derivatives) increases the repertoire of antibiotic drugs. Thus, at least in E. coli and probably in other bacteria as well, bacteriostatic antibiotics could be turned into bactericidal antibiotics by using EDF to turn on the built-in *mazEF* system.

## Materials and Methods

### Bacterial strains and plasmids.

The following strains were used: MC410*relA^+^* and its Δ*mazEF*::*kan* derivative [43]; E. coli strain K38 and its Δ*mazEF* derivative [6]; MG1655 [44] and its Δ*mazEF*::*kan* derivative, which we constructed by P1 transduction from strain MC4100*relA1*Δ*mazEF*::*kan*. In addition, we used E. coli strain MC4100*relA1* and its Δ*icdA*, Δ*IscS* and Δ*acnB* derivatives, which we constructed by P1 transduction from the same strains in genetic background of strains BW25113 [3]. We used plasmid pBAD-*mazF* [35]. We constructed plasmids pQE*katE* and pQE*sodA* as follows: *KatE* gene was PCR amplified from strain MG1655 using primers 5′-GGGGTACCCCAGTTCAATGTCGCAACATAACGAAAAG-3′ for sense sequences and 5′-AACTGCAGCCAATGCATTGGAATCCCATCAGGCAGGAATTTTGTCAATC-3′ for antisense sequences. The PCR fragment was digested with *KpnI* and *PstI* restriction enzymes and ligated into the *KpnI-PstI* restriction sites on the multicloning site (MCS) of pQE-30 plasmid (Qiagen) bearing an ampicillin resistance gene, downstream the T5 promoter. *sodA* gene was PCR amplified from strain MG1655 using primers 5′-CGGGATCCCGGATGAATATGAGCTATACCCTGCCATCCCTG-3′for sense sequences and 5′-CCCAAGCTTGGGAAATGATTATTTTTTCGCCGCAA-3′ for antisense sequences. The PCR fragment was digested with *BamHI* and *HindIII* restriction enzymes and ligated into the *BamHI-HindIII* restriction sites on the multicloning site (MCS) of pQE30 plasmid (Qiagen) bearing an ampicillin resistance gene, downstream from the T5 promoter.

### Materials and media.

The bacteria were grown in liquid M9 minimal medium with 1% glucose and a mixture of amino acids (10 μg/ml each) [45] and then plated on rich LB agar plates as described previously [4]. The following materials were obtained from Sigma: IPTG (isopropyl-β-D-thiogalactopyranoside), L-arabinose, nalidixic acid, mitomycin C, trimethoprim, rifampin, serine hydroxamate, chloramphenicol, spectinomycin, 2–2′ dipyridyl, trizma-base, sodium dodecyl sulphate (SDS), DNAse, and RNAse. We also used the following materials: lysosyme and glycerol (United States Biochemical Corporation), and ampicillin (Biochemie GmbH). Carbonylated proteins were detected using the chemical and immunological reagents from the OxyBlot Oxidized Protein Detection Kit (Chemicon), nitrocelloluse membranes (Pall Corporation). The chemiluminescence assay was performed using luminol, and p-cumaric acid (Sigma) and hydrogen peroxidase solution (Merck), AnaeroGen bags (Gamidor Diagnostics). Chemically synthesized EDF and iEDF peptides (having 98% purity) were synthesized for us by GenScript Corporation.

### Determining the effect of aerobic and anaerobic conditions on *mazEF*-mediated cell death.

Aerobic conditions. Cells were grown in M9 medium with shaking (160 rpm) at 37 °C for 12 h. Then, cells were diluted 1:100 in 10 ml of M9 medium and were grown with shaking (160 rpm) at 37 °C to mid-logarithmic phase (OD_600_ 0.6). The cells were grown in 50-ml tubes. Samples of 500 μl were withdrawn into Eppendorf tubes (1.5-ml volume) and were further incubated without shaking at 37 °C for 10 min as described below for each case. Stressful conditions were induced as described in each figure legend. The cells were centrifuged and re-suspended in pre-warmed saline, diluted, plated on pre-warmed LB plates, and incubated at 37 °C for 12 h. Cell survival was calculated by comparing the number of the colony-forming units of cells treated by stressful conditions to those of the cells that were not exposed to the treatment.

Anaerobic conditions. Cells were grown in 15-ml tubes containing 10 ml of M9 medium standing without shaking in an anaerobic jar containing AnaeroGen bags at 37 °C. The cells were incubated for 10–12 h until the cultures reached an optical density of OD_600_ of 0.6. Samples of 1 ml were withdrawn into 1.5 ml Eppendorf tubes and were further incubated at 37 °C by standing in the anaerobic jar for 10 min. Then, stressful conditions were induced under anaerobic conditions as described in the legend to Figure 4. The cells were centrifuged, washed, diluted, and plated as described above and incubated in the anaerobic jar at 37 °C for 20 h. Cells survival was calculated as described above.

### Determining the effect of inhibition of ROS formation on E. coli cell death.

*Addition of catalase or 2–2′ dipiridyl (iron chelator).* Cells were grown in aerobic conditions as described above. The cells were incubated without shaking at 37 °C with or without catalase (20 μg/ml) or 2–2′ dipiridyl (1mM) for 10 min. Then, stressful conditions were induced as described in the figure legends. The cells were centrifuged and washed in pre-warmed saline with or without catalase as described above. The cells were diluted in pre-warmed LB, plated on pre-warmed LB plates and incubated at 37 °C for 12 h. Cells survival was calculated as described above.

*Over-expression of katE and sodA.* Cells were transformed with plasmid pQE*katE* or pQE*sodA*. The strains were grown in M9 medium with ampicillin (100 μg/ml) in aerobic conditions as described above, and incubated for 10 min without shaking. For the rest of the experiment, see the previous paragraph.

### Determining the effect of MazF overproduction.

Cells were grown in M9 minimal medium containing 0.5% glycerol as a carbon source with ampicillin (100 μg/ml) and chloramphenicol (50 μg/ml) in aerobic conditions or in anaerobic conditions as described above. Then 0.2% arabinose was added in order to induce *mazF* expression and cultures were incubated at 37 °C without shaking for 1 h. Then 0.2% glucose was added. The cultures were incubated in M9 for additional 8 h in aerobic conditions or anaerobic conditions as described above.

### Determination of the cellular level of protein carbonylation.

Cells were grown in M9 medium and the cultures were submitted to specific stressful conditions for the time required to induce the expression of the chromosomally borne *mazEF* module as described above. After the induction of *mazEF*, the agents added to cause stressful conditions were removed by centrifugation. Then the cells were washed, and re-suspended in M9 medium preheated to 37 °C and further incubated. At various times, cells were lysed as follows: 1 ml of the culture was washed with 50 mM Tris buffer (pH 7.5) and centrifuged for 10 min at 14,000 rpm. The pellet was re-suspended in 150 μl lysis buffer containing 0.5 mg/ml lysosyme, 20 μg/ml DNAse, 50 μg/ml RNAse, 1 mM EDTA, and 10 mM Tris (pH 8). 15μl of 10% SDS solution was added and the cells were incubated at 100 °C for 5 min. To examine the level of protein carbonylation in these lysates, we used the Chemicon OxyBlot kit to derivatize the carbonyl groups in the protein side chains to 2,4-dinitrophenylhydrazone (DNP-hydrazone) by reaction with 2,4-dinitrophenylhydrazine. These DNP-derivative crude protein extracts were dot blotted onto nitrocellulose membrane, which was incubated with primary antibody, specific to the DNP moiety of the proteins, and subsequently incubated with secondary (goat anti-rabbit) horseradish peroxidase-antibody conjugate directed against the primary antibody. Carbonylation was observed by ECL. The intensity of each band was quantified using the Image Master VPS-CL (Amersham Pharmacia Biotec). In the case of a poor signal, the samples were concentrated by speed-vac and tested again (see example in Figure S3). In the case of signal saturation the sample was diluted and re-tested. The intensity of each represented band was normalized to equal levels of protein which were determined using Bradford reagent (Bio-Rad).

### Determining the effect of mazEF and EDF on the mode of action of rifampicin or nalidixic acid.

*E. coli* strains MC4100*relA* ^+^, MC4100*relA* ^+^ *mazEF*, and MG1655 were either transformed with plasmid pQE*katE* or pQE*sodA* or not transformed. Then, cells were grown aerobically as described above. In case of strains MG1655/pQE*katE* and MG1655/pQE*sodA*, cells were grown with IPTG (1 mM). Mid-logarithmic cells were applied with catalase or 2–2′-dipyridyl or EDF (0.05 μg/ml ) or iEDF (0.05 μg/ml ) and incubated for 10 min without shaking at 37 °C. Rifampicim (20 μg/ml) or nalidixic acid (1 mg/ml) were added and cells were further incubated. For CFU/ml measurements, 100 μl of each culture was collected at different time points. The cells were centrifuged and re-suspended in pre-warmed saline, diluted in pre-warmed LB, and plated on LB plates as described above.

## Supporting Information

Figure S1 The Effect of Deletion of *iscS* on Cell Death Triggered by Various Stressful Conditions E. coli MC4100*relA*1 (WT) and MC4100*relA*1Δ*iscS* (Δ*iscS*) were grown aerobically to mid-log phase. Cells were incubated without shaking at 37 °C for 10 min, followed by the addition of either rifampicin (20 μg/ml) (Rif), spectinomycin (1 mg/ml) (Spec), or nalidixic acid (Nal) for 10 min. Error bars indicate standard deviation.
(275 KB EPS)

Figure S2 The Effect of Deleting Genes Specifying for TCA Cycle Components on Cell Death Triggered by Rifampicin E. coli MC4100*relA*1 (WT), MC4100*relA*1Δ*icdA* (Δ*icdA*), and MC4100*relA*1Δ*acnB* (Δ*acnB*) were grown aerobically to mid-log phase. Cells were incubated without shaking at 37 °C for 10 min, followed by the addition of rifampicin (20 μg/ml) for 10 min. Error bars indicate standard deviation.
(262 KB EPS)

Figure S3 Determining the Basal Level of Protein Carbonylation in E. coli
E. coli strains MC4100*relA* ^+^WT and its Δ*mazEF* derivative were grown to mid-logarithmic phase as described in Materials and Methods. Cell lysates were prepared and carbonylation was observed by ECL as described in Materials and Methods. Due to a poor signal, the samples were concentrated by speed-vac (the multiplicity of concentration is stated above each band) and the sample was tested again. This procedure allowed quantifying the intensity of apparent bands which were determined by Image Master VPS-CL (Amersham Pharmacia Biotec).
(536 KB JPG)

Figure S4 *mazEF*-Mediated Carbonylation of Cellular Protein Following Various Stressful Conditions (A, C, and E) Protein carbonylation: E. coli strains MC4100*relA* ^+^ (WT) and its derivative MC4100*relA* ^+^Δ*mazEF* (Δ*mazEF*) were grown as described in Materials and Methods. Stressful conditions were induced by incubation of the cells at 37 °C without shaking with: (A) Rifampicin (20 μg/ml) for 10 min. (C) Nalidixic acid (1 mg/ml) for 10 min. (E) Trimethoprim (2 μg/ml) for 1h. Protein carbonylation was determined as described in Materials and Methods.
(B, D, and F) Relative carbonyl levels described in (A, C, and E). The intensity of each band observed in (A, C, and E) was quantified as described in Materials and Methods. The numbers express the relative carbonyl levels of each treated strain compared with that of the untreated WT strain, which has been determined to be one (1).
(926 KB EPS)

Figure S5 Determining the Effect of MazF Overproduction on ROS Formation (A and B) and Cell Survival (C) E. coli strains MC4100*relA* ^+^pBAD-*mazF* (MazF) was grown in M9 minimal medium in aerobic or anaerobic conditions as described in Materials and Methods.
(A) Protein carbonylation under aerobic conditions; (B) Protein carbonylation under anaerobic conditions, positive control of DNP-derivatized proteins was provided by the Chemicon OxyBlot kit; (C) Percent (%) of survivors was determined 8 h following the induction of *mazF* expression.
(372 KB EPS)

## Abbreviations

EDF: Extracellular Death Factor
ROS: Reactive Oxygen Species
TA: toxin–antitoxin
WT: wild type
