# Expression of Concern: The Communication Factor EDF and the Toxin–Antitoxin Module *mazEF* Determine the Mode of Action of Antibiotics

**DOI:** 10.1371/journal.pbio.3001246

**Published:** 2021-05-10

**Authors:** The PLOS Biology Editors

**Affiliations:** Public Library of Science, San Francisco, California, United States of America and Cambridge, United Kingdom

After this article [1] was published, concerns were raised about results reported in Figures 2, 3, S3, S4, and S5. PLOS followed up with the authors who provided available data from the original experiments and replication experiments.

The concerns, authors’ responses, and editorial assessment of materials provided post-publication are outlined here:

1. **The same WT results appear to be reported in Figure 2A-B and Figure S4A-B**. The figure legends report the same treatment conditions for Figure 2A and S4A, and the authors noted that these panels were intended to report distinct technical replicates of the same experiment. Image data provided to the journal indicated that for Figure 2A, either mutant data were not obtained on the same blot as the WT data shown, or the wrong data were shown in the mutant panel. Nevertheless, the data provided support the results shown in Figures 2A and S4A.
2. **Similarities were noted between the WT panels in Figures 2C, 2E, and S4D, when the aspect ratio is adjusted for Figure 2C**. The authors provided original data supporting Figures 2C, 2E, and S4D (S1 File, left and middle blots) and stated that Figure 2C in [1] reports different WT data than Figure 2E and S4D. Per the Editors’ assessment, there are differences between the published Figure 2C and the supporting blot image provided for this figure. The underlying data provided for Figure 2E and S4D indicate that the results shown in these figures were obtained in the same experiment (trimethoprim treatment, 2μg/ml, 1h) that included WT, Δ*mazEF*, and catalase (20μg/ml and 100μg/ml) treated samples, but not KatE samples. The reuse of WT data in Figure 2E and S4D was therefore valid but should have been indicated in the figure legends. (Additional concerns about the KatE and WT+Catalase (100μg/ml) panels of Figure S4D are discussed below in point 4.)
  The image provided for the WT-Catalase (20μg/ml) blot in Figure S4D (S1 File) did not include clearly discernible blot data for the 0 timepoint. The authors disagreed with this assessment and noted that the T0 timepoint sample was taken before Catalase was applied so the same condition is represented by T0 data for untreated samples.
  Also, the supporting image data for Figure S4D include one film fragment for WT+Catalase (20μg/ml) data and a second film fragment with data for other conditions (middle blots in S1 File). As an image of the full-sized, intact and uncropped film was not provided we cannot clarify whether the two film fragments were generated in the same exposure and/or originated from the same blot. Quantitative comparisons of the WT+Catalase (20μg/ml) vs. other groups in Figure S4D should be interpreted with caution in light of this issue. The authors commented that the two film fragments were pasted to the same page of the lab notebook and that this provides support for the data having originated from the same blot. They also provided additional replication data to support Figure S4D (S1 File, right-most blot).
3. **The MazF panels in Figures 3A and S5A appear to report the same data**. The experiment in Figure 3A was done under aerobic conditions and so these panels represent the same experimental conditions. Reuse of the same WT panel and splicing of the blot image between the 1 hour and 2 hour samples should have been explained in the figure legends. Per data received from the authors, the journal confirmed that the data within this panel were run on the same blot, and that these figures accurately represent the relative intensity of the bands observed on the underlying images. Based on image data provided to the journal, it is unclear whether MazF and MazF+KatE results were obtained from the same blot and/or exposure.
4. **Concerns were raised about image data reported in Figures S3 and S4**:
  - The Figure S3 ‘WT-baseline’ and Figure S4D ‘WT-Catalase (20μg/ml)’ panels appear similar. Slots 1–3 in these panels also appear similar to slots 1–3 in the Figure S3 ‘Δ*mazEF*-base line’ panel.
  - Figure S3 ‘Δ*mazEF*-base line’ panel slots 4–5 and Figure S4D ‘WT’ panel slots 2–3 appear similar.
  - When brightness and/or contrast levels are adjusted there appear to be background discontinuities in both Figure S3 panels and in the WT and WT+Catalase (20μg/ml) panels of Figure S4D.
  - In Figure S4A, when contrast levels are adjusted, the WT+KatE panel and the lower half of the WT+Catalase (20μg/ml) panel appear to be devoid of image content or include a solid white box.
  - In Figure S4D, when levels are adjusted, the ‘WT+Catalase(100μg/ml)’ and ‘WT+KatE’ panels appear similar and include handwritten markings but no clear evidence of experimental blot data.
  The authors noted that the original data underlying Figure S3 are no longer available. They acknowledged the poor quality of Figure S3, provided the journal replication data for the Figure S3 results, and commented that this figure reports a calibration experiment that is not crucial for supporting the article’s main claims.
  As noted above, original image data were provided in support of Figure S4D. [See S1 File and comments under point 2 regarding the WT-Catalase (20μg/ml) blot data.]
  The data provided to the journal (including those in S1 File) lend support for the main claims made in the article based on Figures S3 and S4D. However, the data did not resolve the concerns about the published figures.
5. **The Figure S4 legend does not correctly describe the figure**. The published figure does not report data for the corresponding nalidixic acid experiment. The corrected figure legend is provided here:
  **Figure S4. *mazEF*-Mediated Carbonylation of Cellular Protein Following Various Stressful Conditions For The Corresponding Trimethoprim Experiment**.
  Original data for Trimethoprim indicate that WT control data were not included on the film fragment showing results of the low concentration catalase fragment. The KatE experiments ran with their own control. E. coli strain MC4100re/A+ (WT) either carrying KatE or not was grown as described in Materials and Methods. Stressful conditions were induced by incubation of the cells at 37 °C without shaking with: (A-C) Rifampicin (20 μg/ml) for 10 min, or (D-F) Trimethoprim (2 μg/ml) for 1h. Catalase was applied at T = O at indicated concentrations (20μg/ml if indicated, or 100μg/ml) or KatE was induced by IPTG 1mM (A, D). Protein carbonylation was determined as described in Materials and Methods (B, E). The intensity of each band observed in (A, D) was quantified as described in Materials and Methods from three independent repeats. The numbers express the relative carbonyl levels of each treated strain compared with the average untreated WT strain, which has been set to one (1). (C, F) Survival assays were performed as indicated and published previously (Kolodkin-Gal et al., Science, 2007, at indicated intervals).

The corresponding author’s institution appointed a faculty member to review the concerns raised about this work and the original laboratory records for the experiments in question. Following their assessment, the reviewer concluded that the data supported the conclusions reported in the article, and that serious errors were made in compiling figures but they did not find evidence of deliberate misconduct or misleading data in the article.

The reviewer also noted that the wrong data were reported in Figure S3 and the wrong control data were reported in Figure S4D, but the authors disagreed with these comments.

The *PLOS Biology* Editors concluded that the main conclusions of the article appear to be supported by the data provided in post-publication discussions. However, we issue this Expression of Concern due to the extent of image issues identified in [1] and the nature of the issues raised for Figures S3 and S4 that remain unresolved. The authors acknowledge the potential errors during the assembly of Figures S3 and S4.

The available original and replicate data to support the article’s results, including those discussed above, can be requested from the corresponding author.

## Supporting information

S1 File Image data provided to support Figures 2C, 2E, and S4D. Blots on the left and in the middle represent the original data supporting the reported results, and the blot on the right is from a replicate experiment for Figure S4D.
(PDF)
