# Supplementary figures and images for: Expression of Concern: The Communication Factor EDF and the Toxin–Antitoxin Module mazEF Determine the Mode of Action of Antibiotics
Source: PLoS Biol. 2021 May 10;19(5):e3001246. doi: 10.1371/journal.pbio.3001246 (PMC8109820; doi:10.1371/journal.pbio.3001246)

Fig 2C raw blot image

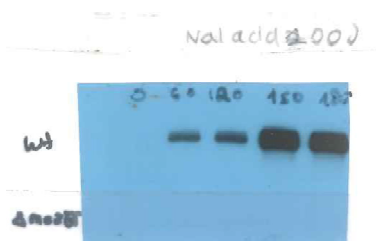

Fig 2E/S4D raw blot image

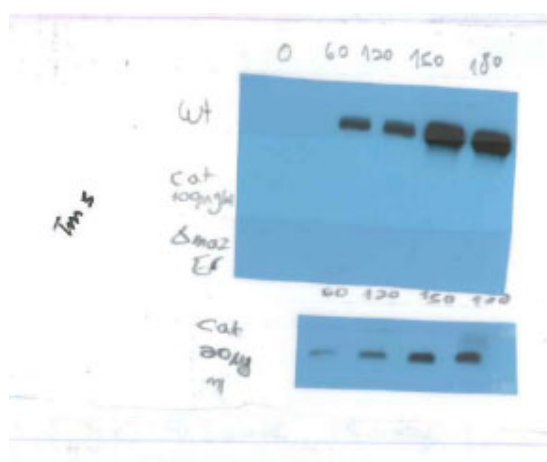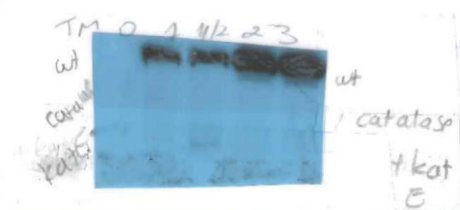

Supplement: S1 File — Blots on the left and in the middle represent the original data supporting the reported results, and the blot on the right is from a replicate experiment for Figure S4D. (PDF) [file pbio.3001246.s001.pdf]
